# Blackcurrant Suppresses Metabolic Syndrome Induced by High-Fructose Diet in Rats

**DOI:** 10.1155/2015/385976

**Published:** 2015-10-04

**Authors:** Ji Hun Park, Min Chul Kho, Hye Yoom Kim, You Mee Ahn, Yun Jung Lee, Dae Gill Kang, Ho Sub Lee

**Affiliations:** ^1^Hanbang Body-Fluid Research Center, Wonkwang University, Shinyong-dong, Iksan, Jeonbuk 570-749, Republic of Korea; ^2^College of Oriental Medicine and Professional Graduate School of Oriental Medicine, Wonkwang University, Shinyong-dong, Iksan, Jeonbuk 570-749, Republic of Korea; ^3^Brain Korea (BK) 21 Plus Team, Professional Graduate School of Oriental Medicine, Wonkwang University, Iksan, Jeonbuk 540-749, Republic of Korea

## Abstract

Increased fructose ingestion has been linked to obesity, hyperglycemia, dyslipidemia, and hypertension associated with metabolic syndrome. Blackcurrant (*Ribes nigrum*; BC) is a horticultural crop in Europe. To induce metabolic syndrome, Sprague-Dawley rats were fed 60% high-fructose diet. Treatment with BC (100 or 300 mg/kg/day for 8 weeks) significantly suppressed increased liver weight, epididymal fat weight, C-reactive protein (CRP), total bilirubin, leptin, and insulin in rats with induced metabolic syndrome. BC markedly prevented increased adipocyte size and hepatic triglyceride accumulation in rats with induced metabolic syndrome. BC suppressed oral glucose tolerance and protein expression of insulin receptor substrate-1 (IRS-1) and phosphorylated AMP-activated protein kinase (p-AMPK) in muscle. BC significantly suppressed plasma total cholesterol, triglyceride, and LDL content. BC suppressed endothelial dysfunction by inducing downregulation of endothelin-1 and adhesion molecules in the aorta. Vascular relaxation of thoracic aortic rings by sodium nitroprusside and acetylcholine was improved by BC. The present study provides evidence of the potential protective effect of BC against metabolic syndrome by demonstrating improvements in dyslipidemia, hypertension, insulin resistance, and obesity *in vivo*.

## 1. Introduction

Metabolic syndrome is a disease condition characterized by variable coexistence of obesity, hyperuricemia, hyperinsulinemia, hypertension, and dyslipidemia. The pathogenesis of metabolic syndrome includes multiple organs in the cardiorenal system [1]. Patients with metabolic syndrome, as defined by the National Cholesterol Education Program Adult Treatment Panel III (NCEP-ATP III), simultaneously exhibit 3 or more of the following traits: increased waist circumference, elevated blood pressure, reduced high-density lipoprotein (HDL) level, elevated triglyceride level, and hyperglycemia [2, 3].

Fructose, present in added sugars such as sucrose and high-fructose corn syrup, has been epidemiologically linked with metabolic syndrome. Increased consumption of fructose, commonly used in processed food and soft drinks, is one of the most important factors contributing to the growing prevalence of metabolic syndrome [4]. Recent findings have shown that dietary fructose accelerates metabolic disorders and induces oxidative damage [5]. A high-fructose diet induces a well-characterized metabolic syndrome, generally resulting in hyperinsulinemia, hypertension, dyslipidemia, and a low HDL level [6]. A recent study suggested that renal damage is associated with metabolic syndrome [7]. Exposure of the liver to high levels of fructose leads to rapid stimulation of lipogenesis and triglyceride accumulation, which lead to reduced insulin sensitivity and hepatic insulin resistance/glucose intolerance [8].

Blackcurrant (*Ribes nigrum*; BC) is a valuable horticultural crop in Russia, Poland, German, Scandinavia, England, New Zealand, and several Eastern European nations. Annual worldwide production of BC is approximately 500,000 to 600,000 tons [9]. BC contains numerous physiologically active components, including vitamins, carotenoids, and flavonoids, which have antiulcer, anticonvulsion, and antidiarrhea effects, as well as protective effects against carcinomas, diabetes, and regressive disease [10, 11].

Several studies have reported that BC produces antioxidative effects. Anthocyanins from BC abrogate oxidative stress through Nrf2-mediated antioxidant mechanisms [12]. In addition, BC extract has cytoprotective and anti-inflammatory properties [13]. Moreover, BC protects against kidney stones [14]. However, the therapeutic effects of BC in subjects with metabolic disorder have not been reported. Thus, this study was designed to identify the effect of BC extract on high-fructose diet-induced metabolic syndrome in rats.

## 2. Methods and Materials

### 2.1. Plant Material and Preparation of BC Extract

BC was purchased from Gukmin Farm (Jeongeup, Korea). A voucher specimen (number HBF191) was deposited in the Herbarium of the Professional Graduate School of Oriental Medicine, Wonkwang University (Korea). BC (400 g) was boiled with 3 L of distilled water at 100°C for 2 h. The resulting extract was filtered through Whatman No. 3 filter paper and centrifuged at 990 ×g for 20 min at 4°C. The resulting supernatant was concentrated using a rotary evaporator, after which the resulting extract (63.19 g) was lyophilized using a freeze-drier and stored at −70°C until required.

### 2.2. Animal Experiments and Diet

All experimental procedures were carried out in accordance with the National Institute of Health Guide for the Care and Use of Laboratory Animals and were approved by the Institutional Animal Care and Utilization Committee for Medical Science of Wonkwang University (approval number: WKU 14-50). Six-week-old male Sprague-Dawley (SD) rats were obtained from Samtako (Osan, Korea) and kept in a room with automatically maintained temperature (23°C), humidity (50–60%), and light/dark cycles (12-h each) throughout the experiments. After 1 week of acclimatization, the rats were randomly divided into 4 groups (10 rats per group). The control group (Cont.) was fed a regular diet. The high-fructose (HF) diet group was fed a 60% fructose diet (Research Diet, USA). The low-dose BC group was fed 60% fructose diet with 100 mg/kg/day BC administered orally by Sonde for a period of 4 weeks. The high-dose BC group was fed 60% fructose diet with 300 mg/kg/day of BC administered orally by Sonde for a period of 4 weeks. The regular diet was composed of 50% starch, 21% protein, 4% fat, and a standard mix of vitamins and minerals. The high-fructose diet was composed of 60% fructose, 20% protein, 10% fat, and a standard mix of vitamins and minerals.

### 2.3. Measurement of Blood Pressure

Systolic blood pressure (SBP) was determined using noninvasive tail-cuff plethysmography and recorded with an automatic sphygmograph (MK2000; Muromachi Kikai, Tokyo, Japan). SBP was measured once per week. At least 10 determinations of SBP were made during every measurement session. Values are presented as the mean ± SEM of 8 measurements.

### 2.4. Estimation of Oral Glucose Tolerance

Two oral glucose tolerance tests (OGTT) were performed 2 days apart after 8 weeks of treatment. Briefly, basal blood glucose concentrations were measured after 10–12 h of overnight fasting, after which glucose (2 g/kg body weight) was immediately administered via oral gavage. Tail vein blood samples were collected 30, 60, 90, and 120 min after glucose administration.

### 2.5. Blood and Tissue Sampling

At the end of the experiments, the thoracic aorta and muscle were separated, rinsed with cold saline, and frozen. Plasma was obtained from coagulated blood samples by centrifugation at 3,000 rpm for 15 min at 4°C and frozen at −80°C.

### 2.6. Blood Parameters

Triglyceride levels in plasma were measured using commercial kits (AM 157S-K, ASAN, Korea). Levels of HDL, total cholesterol, and low-density lipoprotein (LDL) in plasma were measured using HDL and LDL assay kits (E2HL-100, Bio Assay Systems, Germany). Levels of insulin in plasma were measured using commercial kits (80-INSRT-E01, ALPCO, UK). Levels of C-reactive protein (CRP) in plasma were measured using commercial kits (557825, BD Biosciences, America). Levels of leptin in plasma were measured using commercial kits (ab100773, Abcam, UK). Levels of T-Bill and BUN in plasma were measured using commercial kits (77184, Arkray, Japan).

### 2.7. Preparation of Aorta and Measurement of Vascular Reactivity

The thoracic aorta was rapidly and carefully collected from each rat and placed into cold Kreb's solution (118 mM NaCl, 4.7 mM KCl, 1.1 mM MgSO_4_, 1.2 mM KH_2_PO_4_, 1.5 mM CaCl, 25 mM NaHCO_3_, 10 mM glucose, and pH 7.4). Connective tissue and fat were removed from each thoracic aorta. Each thoracic aorta was cut into rings of approximately 3 mm in length. Care was taken to protect the endothelium from accidental damage during the dissection procedure. The thoracic aortic rings were suspended in a tissue bath containing Kreb's solution at 37°C by means of 2 L-shaped stainless-steel wires inserted into the lumen and aerated with 95% O_2_ and 5% CO_2_. The isometric forces of the rings were measured using a Grass FT 03 force displacement transducer connected to a Model 7E polygraph recording system (Grass Technologies, Quincy, MA, USA). A passive stretch of 1 g in the thoracic aortic rings was determined to be the optimal tension for maximal responsiveness to phenylephrine (10^−6^ M). The preparations were allowed to equilibrate for approximately 1 h with the Kreb's solution replaced every 10 min. The relaxant effects of acetylcholine (ACh, 10^−9^–10^−6^ M) and sodium nitroprusside (SNP, 10^−10^–10^−5^ M) in the thoracic aorta rings were studied.

### 2.8. Western Blot Analysis in the Rat Aorta and Muscle

Thoracic aorta and muscle homogenates were prepared in ice-cold buffer containing 250 mM sucrose, 1 mM EDTA, 0.1 mM phenylmethylsulfonyl fluoride, and 20 mM potassium phosphate buffer (pH 7.6). The homogenates were centrifuged at 8,000 rpm for 10 min at 4°C, after which the resulting supernatants were centrifuged at 13,000 rpm for 5 min at 4°C to produce a cytosolic fraction for protein analysis. The recovered proteins were separated by 10% SDS-polyacrylamide gel electrophoresis and transferred to nitrocellulose membranes, which were blocked with 5% bovine serum albumin (BSA) powder in 0.05% Tween 20-Tris-buffered saline (TBS-T) for 1 h. Specific primary antibodies against ICAM-1, VCAM-1, E-selectin, eNOS, ET-1 (in aorta), AMP-activated protein kinase (AMPK), p-AMPK, and insulin receptor substrate-1 (IRS-1) (in muscle) were purchased from Santa Cruz Biotechnology, Inc. (Santa Cruz, CA, USA). The nitrocellulose membranes were incubated overnight at 4°C with protein antibodies. The blots were washed several times with TBS-T and incubated with horseradish peroxidase-conjugated secondary antibody for 1 h after which immunoreactive bands were visualized using an enhanced chemiluminescence system (Amersham, Buchinghamshire, UK). The bands were analyzed densitometrically using a Chemi-doc Image Analyzer (Bio-Rad, Hercules, CA, USA).

### 2.9. Hematoxylin and Eosin (H & E) and Oil Red O Staining of Aorta, Epididymal Fat, and the Liver

Thoracic aorta tissue from 5 random subjects from each group was fixed in 10% (v/v) formalin in 0.01 M phosphate-buffered saline (PBS) for 2 days, with the formalin solution changed every day to remove traces of blood. The aortic tissue samples were dehydrated and embedded in paraffin, sectioned (6 *μ*m), and stained with H & E. Epididymal fat (from 5 random subjects from each group) and liver tissue (from 5 random subjects from each group) samples were fixed in 4% paraformaldehyde for 48 h at 4°C and incubated with 30% sucrose for 2 days. Each fat and liver sample was embedded in OCT compound (Tissue-Tek, Sakura Finetek, Torrance, CA, USA), frozen in liquid nitrogen, and stored at −80°C. Frozen sections were cut with a Shandon Cryotome SME (Thermo Electron Corporation, Pittsburg, PA, USA) and mounted on poly-l-lysine-coated slides. Epididymal fat sections were stained with H & E, whereas liver sections were stained with Oil Red O. For quantitative histopathological comparisons, each section was analyzed using Axiovision 4 Imaging/Archiving software.

### 2.10. Immunohistochemistry of Thoracic Aorta Tissue

Prior to immunohistochemical staining, tissue sections in paraffin were mounted on poly-l-lysine-coated slides (Fisher Scientific, Pittsburgh, PA, USA). The tissue on each slide was immunostained using Invitrogen HISOTO-STAIN-SP kits with the labeled streptavidin-biotin (LAB-SA) method. After antigen retrieval, slides were immersed in 3% hydrogen peroxide for 10 min at room temperature to block endogenous peroxidase activity and rinsed with PBS. Next, slides were incubated with 10% nonimmune goat serum for 10 min at room temperature and incubated with primary antibodies against ICAM-1, VCAM-1, ET-1, and eNOS (1 : 200; Santa Cruz, CA, USA) in humidified chambers overnight at 4°C. Next, slides were incubated with biotinylated secondary antibodies for 20 min at room temperature, followed by incubation with horseradish peroxidase-conjugated streptavidin for 20 min at room temperature. Peroxidase activity was visualized using a 3,3′-diaminobenzidine (DAB; Novex, CA) substrate-chromogen system with hematoxylin counterstaining (Zymed, CA, USA). For quantitative analysis, the average score of 10–20 randomly selected areas was calculated using NIH Image Analysis Software, ImageJ (NIH, Bethesda, MD, USA).

### 2.11. Statistical Analysis

All experiments were repeated at least 3 times. Results are expressed as mean ± S.D. or mean ± S.E.M. Data were analyzed using Sigmaplot 10.0 software. Student's *t*-test was used to determine significant differences between groups. Results of *P* < 0.05 were considered statistically significant.

## 3. Results

### 3.1. Effects of BC on Body Weight and Tissue Weights

During the experimental period, all groups showed significant increases in body weight. The body weight of the HF diet group was not significantly different from that of the control group. However, the BC1 and BC2 groups showed significantly decreased body weight in comparison with the HF diet group during the final week of the experiment.

In comparison with the control group, the HF diet group showed significantly increased liver weight, liver weight as a percentage of BW, epididymal fat pad weight, and epididymal fat pad weight as a percentage of BW. However, the BC1 and BC2 groups showed significantly reduced liver weight, liver weight as a percentage of BW, epididymal fat pad weight, and epididymal fat pad weight as a percentage of BW (Table 1).

### 3.2. Effects of BC on Plasma Parameters

The HF diet group showed significantly increased CRP, T-Bil, and insulin levels in comparison with those of the control group. However, the BC2 group showed significantly decreased CRP, T-Bil, and insulin in comparison with those of the HF diet group. The HF diet group showed significantly decreased leptin in comparison with that of the control group. The BC2 group showed significantly increased leptin in comparison with that of the HF diet group (Table 2).

### 3.3. Effects of BC on Oral Glucose Tolerance and Expression of IRS-1 and p-AMPK in Muscle

Each rat was subjected to the OGTT to measure insulin resistance. The HF diet group showed significantly increased blood glucose content 90 and 120 min after glucose administration. However, the blood glucose content of the BC2 group significantly reduced in comparison with that of the HF diet group 90 and 120 min after glucose administration.

**Figure 1:**
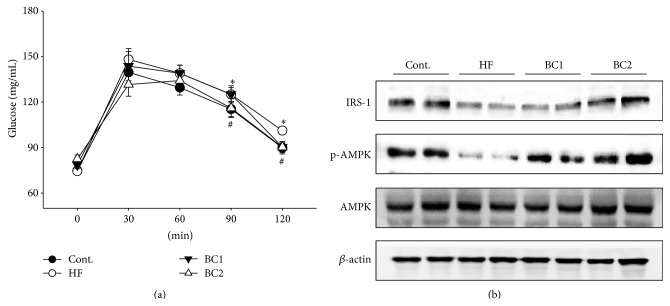
Effects of BC on oral glucose tolerance test (a) and the expression of IRS-1 and p-AMPK in the muscle (b). Values were expressed as mean ± S.E. (*n* = 10). ^*∗*^ *P* < 0.05 versus Cont; ^#^ *P* < 0.05 versus HF. Representative western blots of IRS-1 and p-AMPK protein levels are shown (*n* = 4). IRS-1: insulin receptor substrate 1; Cont.: control; HF: high fructose; BC1: blackcurrant 100 mg/kg/day; BC2: blackcurrant 300 mg/kg/day.

The IRS-1 and p-AMPK protein expression levels of the HF diet group significantly decreased in comparison with those of the control group. However, the IRS-1 and p-AMPK protein expression levels in muscle tissue from the BC1 and BC2 groups reduced in comparison with those of the HF diet group (Figure 1).

### 3.4. Effect of BC on the Morphology of Epididymal Fat Pads

**Figure 2:**
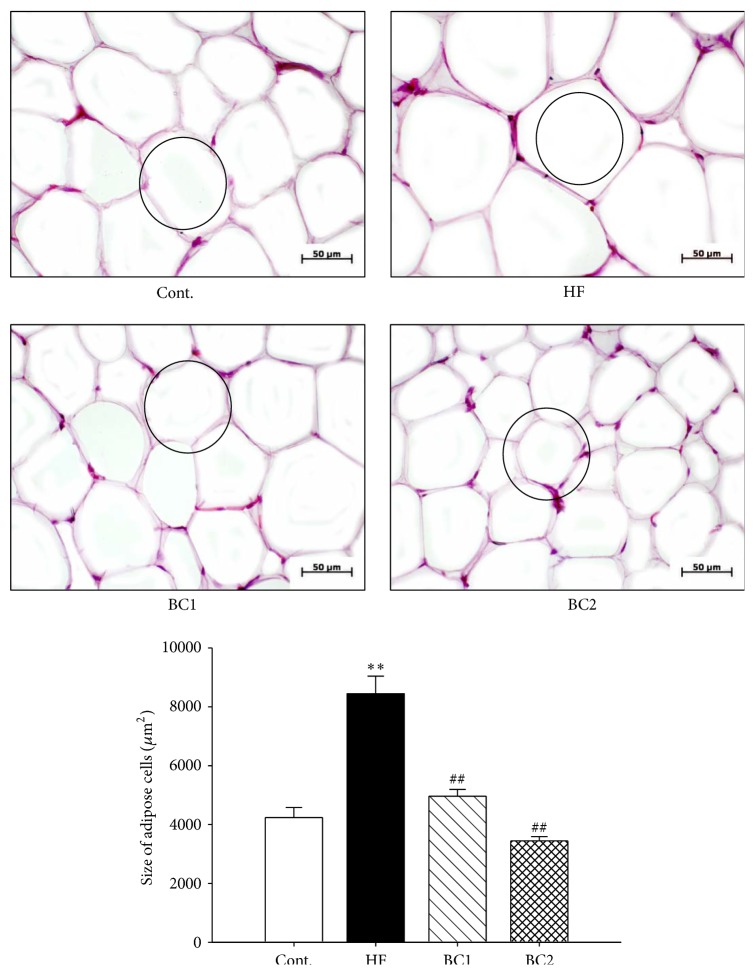
Effects of BC on epididymal fat pads morphology. Representative microscopic photographs in epididymal fat pads of SD rats with control diet and HF diet were stained with hematoxylin and eosin. Values were expressed as mean ± S.E. (*n* = 5). ^*∗∗*^ *P* < 0.01 versus Cont; ^##^ *P* < 0.01 versus HF. Cont.: control; HF: high fructose; BC1: blackcurrant 100 mg/kg/day; BC2: blackcurrant 300 mg/kg/day.

The HF diet induced fat hypertrophy in this study. However, the adipocytes of the BC1 and BC2 groups showed significantly reduced hypertrophy in comparison with those of the HF diet group (Figure 2).

### 3.5. Effect of BC on Hepatic Steatosis

**Figure 3:**
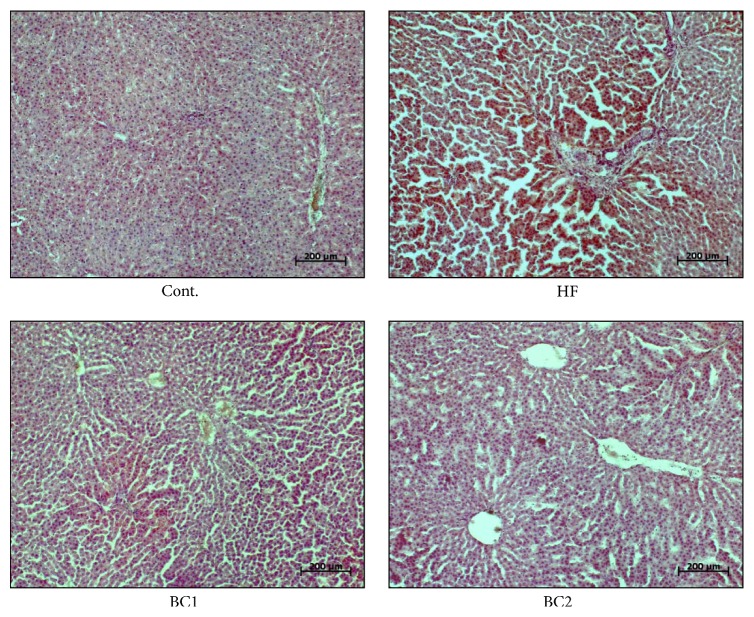
Effects of BC on liver morphology. Representative microscopic photographs in liver of SD rats with control diet and HF diet were stained with Oil red O (*n* = 5). Cont.: control; HF: high fructose; BC1: blackcurrant 100 mg/kg/day; BC2: blackcurrant 300 mg/kg/day.

To investigate fat accumulation in the liver, we prepared frozen liver sections, which were stained with Oil Red O. Lipid droplets were detected in the HF diet groups. However, significantly fewer lipid droplets were counted in the BC1 and BC2 groups compared to those counted in the HF diet group (Figure 3).

### 3.6. Effect of BC on Lipid Metabolism Biomarker Levels

Total cholesterol, triglyceride, LDL, and HDL plasma levels were measured as biomarkers of lipid metabolism. The total cholesterol, triglyceride, and LDL levels of the HF diet group significantly increased in comparison with those of the control group. However, the total cholesterol, triglyceride, and LDL levels of the BC1 and BC2 groups significantly decreased in comparison with those of the HF group. There was no significant difference in the HDL level of either the HF group or the BC group (Table 3).

### 3.7. Effect of BC on Systolic Blood Pressure and Vascular Tension

At the beginning of the experimental feeding period, systolic blood pressure was measured by the tail-cuff technique. After 4 weeks, the systolic blood pressure of the HF diet group was significantly higher than that of the control group. The systolic blood pressure of the BC1 group was significantly lower than that of the HF diet group. The systolic blood pressure of the BC2 group was significantly lower than that of the HF diet group.

**Figure 4:**
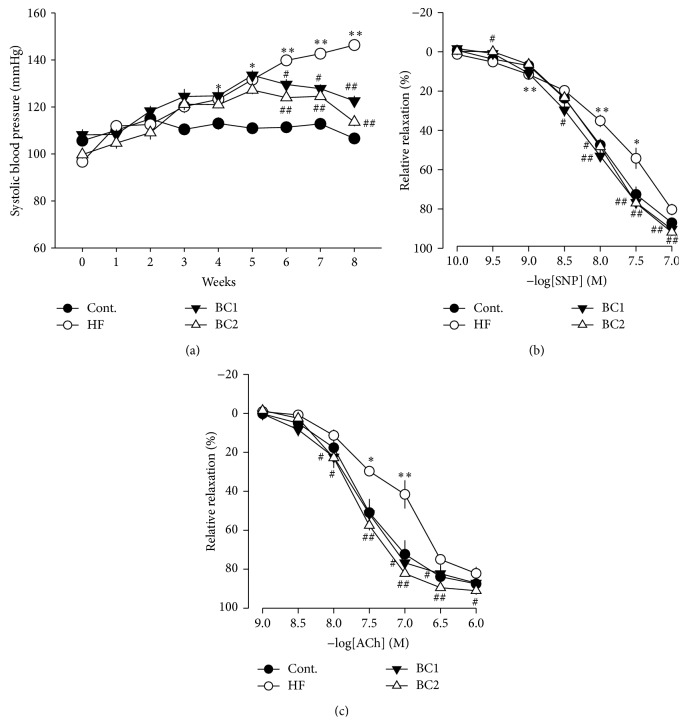
Effects of BC on systolic blood pressure (a), vascular tone in thoracic aorta effect of a BC on sodium nitroprusside-induced contraction in thoracic aorta (b), and effect of a BC on acetylcholine-induced relaxation in thoracic aorta (c). Values were expressed as mean ± S.E. (*n* = 10). ^*∗*^ *P* < 0.05 and ^*∗∗*^ *P* < 0.01 versus Cont.; ^#^ *P* < 0.05 and ^##^ *P* < 0.01 versus HF. SNP: sodium nitroprusside; ACh: acetylcholine; Cont.: control; HF: high fructose; BC1: blackcurrant 100 mg/kg/day; BC2: blackcurrant 300 mg/kg/day.

Vascular responses to SNP (1 × 10^−10^ to 1 × 10^−7^ M) and ACh (1 × 10^−9^ to 1 × 10^−6^ M) were measured in the thoracic aorta. ACh-induced relaxation of thoracic aorta rings was significantly impaired in the HF diet group in comparison with that of the other groups. SNP-induced relaxation of thoracic aorta rings was significantly impaired in the HF diet group in comparison with that of the other groups (Figure 4).

### 3.8. Effect of BC on the Morphology of Aorta

**Figure 5:**
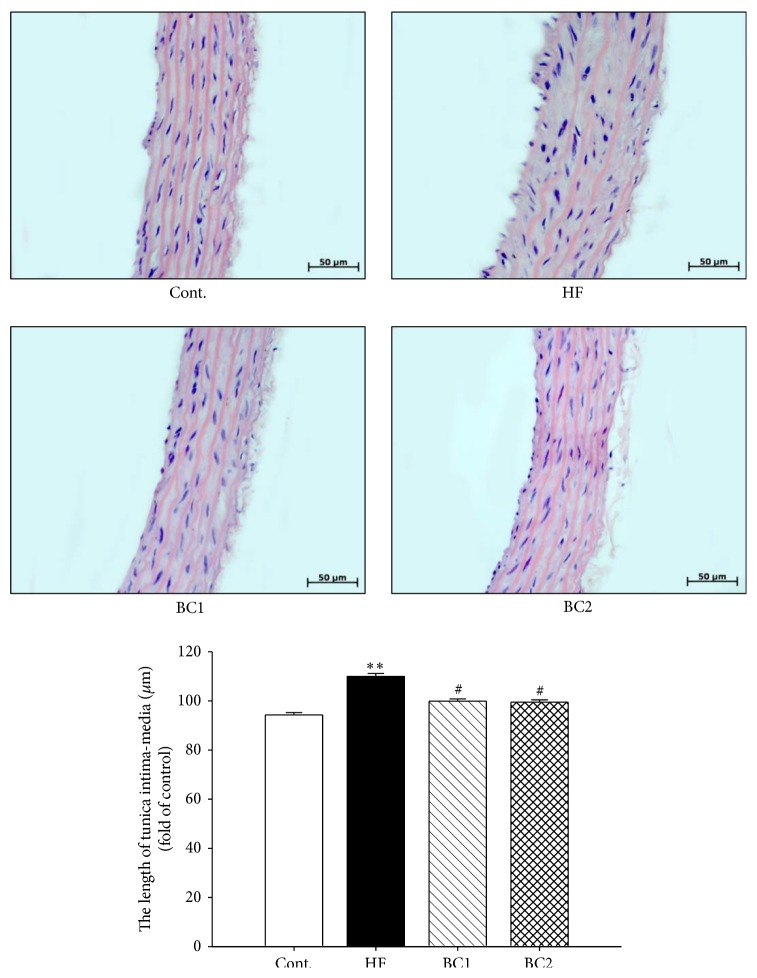
Effects of BC on aorta morphology. Representative microscopic photographs in aorta of SD rats with control diet and HF diet were stained with hematoxylin and eosin. Values were expressed as mean ± S.E. (*n* = 5). ^*∗∗*^ *P* < 0.01 versus Cont.; ^#^ *P* < 0.05 versus HF. Cont.: control; HF: high fructose; BC1: blackcurrant 100 mg/kg/day; BC2: blackcurrant 300 mg/kg/day.

The tunica intima-media layer of the HF diet group showed significantly increased thickness in comparison with that of the control group. However, the tunica intima-media layer of the BC1 and BC2 groups showed significantly decreased thickness in comparison with that of the HF diet group (Figure 5).

### 3.9. Effects of BC on Expressions Levels of Adhesion Molecules, eNOS, and ET-1 in Aortic Tissue

**Figure 6:**
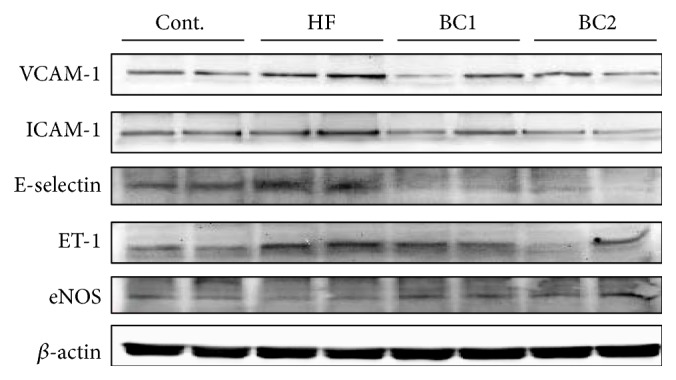
Effect of BC on the expression of adhesion molecules, ET-1, and eNOS in the aorta. Representative western blots of adhesion molecules, eNOS, and ET-1 protein levels are shown (*n* = 5). Cont.: control; HF: high fructose; BC1: blackcurrant 100 mg/kg/day; BC2: blackcurrant 300 mg/kg/day.

Protein expression levels of adhesion molecules (VCAM-1, ICAM-1, and E-selectin), ET-1, and eNOS in aortic tissue were determined by western blotting. Protein levels of adhesion molecules and ET-1 increased in the HF diet group in comparison with those of the control group. However, the BC1 and BC2 groups showed decreased protein levels of adhesion molecules and ET-1 in comparison with those of the HF diet group. In addition, eNOS protein expression in the HF diet group decreased in comparison with that of the control group. However, the BC1 and BC2 groups showed increased eNOS protein expression in comparison with that of the HF group (Figure 6).

### 3.10. Effects of BC on Immunoreactivity of Adhesion Molecules, eNOS, and ET-1 in Aortic Tissues

**Figure 7:**
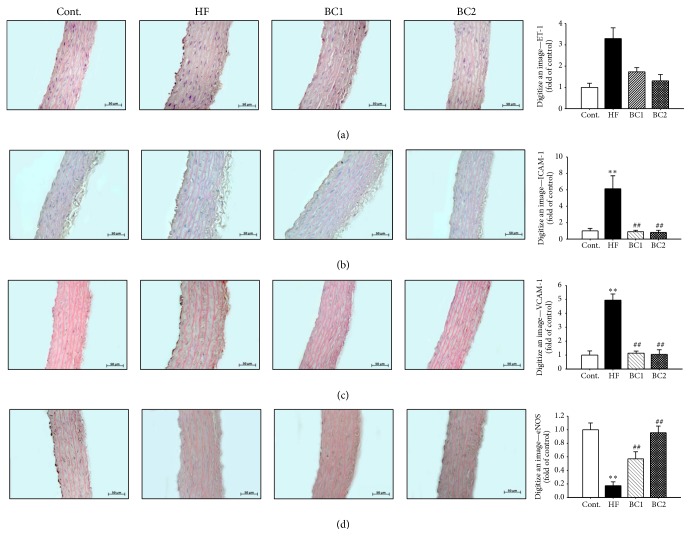
Effects of BC on ET-1 (a), ICAM-1 (b), VCAM-1 (c), and eNOS (d) immunoreactivity in aortic tissues. Values were expressed as mean ± S.E. (*n* = 5). ^*∗∗*^ *P* < 0.01 versus Cont.; ^#^ *P* < 0.05 versus HF. Cont.: control; HF: high fructose; BC1: blackcurrant 100 mg/kg/day; BC2: blackcurrant 300 mg/kg/day.

Immunohistochemistry was used to evaluate expression of ET-1, ICAM-1, VCAM-1, and e-NOS in the aortic wall. ET-1, ICAM-1, and VCAM-1 protein expression levels increased in the HF diet group in comparison with those of the control group. However, the ET-1, ICAM-1, and VCAM-1 protein expression levels of the BC1 and BC2 groups decreased in comparison with those of the HF diet group. eNOS expression decreased in the HF diet group in comparison with that of the control group. However, the groups treated with BC1 and BC2 showed increased eNOS expression in comparison with that of the HF diet group (Figure 7).

## 4. Discussion

The results of this study demonstrate that the HF diet induced metabolic syndrome with increased epididymal fat pad weight resulting from increased plasma levels of triglycerides and LDL. Treatment with BC lowered epididymal fat pad weight, triglyceride levels, and LDL levels, whereas it elevated levels of HDL, which enhances lipid metabolism. Thus, BC improves lipid metabolism by decreasing plasma levels of triglycerides and LDL. Although epididymal fat pad weight increased in response to the HF diet, the body weight of the control diet and HF diet groups was similar [15]. HF has been shown to increase hepatic lipase activity and epididymal fat hypertrophy [7, 16]. Experiments intended to measure increased body weight should be longer than the period of 8 weeks used in the present study [17]. BC reduces obesity in HF diet rats, because BC significantly decreased the HF diet-induced increase in body weight in this study. In addition, BC suppressed insulin and leptin levels [18, 19].

Because HF impairs glucose tolerance and induces obesity, dyslipidemia, and fatty liver, this study focused on the expression of AMPK in the liver and muscle [20]. HF decreased expression of IRS-1. IRS-1 deficiency causes insulin resistance. In addition, IRS-1 plays a very important role in secretion of insulin from pancreatic *β*-cells in the liver and muscle [21]. The BC group showed reduced IRS-1 expression in comparison with that of the HF group.

In the present study, HF increased circulating levels of inflammatory marker CRP. Increased CRP is an independent risk factor for coronary artery disease [22]. Altered lipid levels induced by the HF diet were associated with aortic lesions. Histological analysis demonstrated that the endothelial layers were rougher in aortic sections from HF diet rats, which was associated with a trend towards increased development of atherosclerosis. Intima-media thickness of the thoracic aorta has been shown to correlate with prognosis and the degree of coronary artery disease [23]. BC treatment maintained smooth and soft intima endothelial layers and decreased intima-media thickness in the aortic sections of HF diet rats.

Endothelial dysfunction plays an important role in hypertension, vascular inflammation, other cardiovascular diseases, and metabolic syndrome [24, 25]. In this experimental model, expression of ET-1 and inducible adhesion molecules such as ICAM-1 and VCAM-1 in the arterial wall represented a key event in the development of atherosclerosis. BC ameliorated vascular inflammation by downregulation of ET-1, ICAM-1, VCAM-1, and E-selectin expression in the thoracic aorta. Several studies have shown that reduced blood pressure and endothelial function are related to increased eNOS reactivity, which results in increased production of  NO, a strong vasodilator [26, 27]. In the present study, BC upregulated eNOS levels in the aorta and recovered HF diet-induced impairment of endothelium-dependent vasorelaxation. These results suggest that the hypotensive effect of BC is mediated by endothelium-dependent NO/cGMP signaling. The histological study revealed that BC suppressed vascular inflammation, compatible with the processes of atherosclerosis. In fact, endothelial dysfunction was initially identified as impaired vasodilation in response to specific stimuli such as ACh and bradykinin; therefore, improvement of endothelial function is predicted to regulate lipid homeostasis [28].

## 5. Conclusion

BC improved reduced plasma levels of biomarkers of dyslipidemia, including total cholesterol, triglycerides, and LDL. BC enhanced SNP- and ACh-induced relaxation and suppressed expression of adhesion molecules in the thoracic aorta, reduced systolic blood pressure, and reduced C-reactive protein levels. In addition, BC ameliorated insulin resistance by decreasing insulin release, improving glucose tolerance, and restoring insulin signaling by recovering IRS-1 expression in skeletal muscle tissue. In addition, BC improved obesity parameters such as leptin and adipocyte size. These results suggest that BC ameliorated dyslipidemia, hypertension, insulin resistance, and obesity in rats with HF-induced metabolic syndrome. Taken together, the results of this study demonstrate that BC may be used as a new therapeutic approach for metabolic syndrome.

## Figures and Tables

**Table 1:** Effects of BC on body weight, liver weight, liver weight % of BW, epididymal fat pads weight, and epididymal fat pads weight % of BW.

	Cont.	HF	BC1	BC2
Body weight (g)				
Start	227.62 ± 1.96	221.75 ± 2.64	233.79 ± 1.99	224.27 ± 2.31
Final	466.15 ± 10.75	455.14 ± 8.15	427.14 ± 10.97^#^	419.73 ± 6.6^#^
Liver weight (g)	10.9 ± 0.52	13.2 ± 0.76^*∗*^	11.18 ± 0.21^#^	10.8 ± 0.23^##^
Liver weight % of BW	2.49 ± 0.07	2.8 ± 0.03^*∗*^	2.52 ± 0.03^#^	2.54 ± 0.04^#^
Epididymal fat pads weigh (g)	6.64 ± 0.4	7.47 ± 0.46^*∗*^	6.42 ± 0.55^#^	5.15 ± 0.42^#^
Epididymal fat pads weight % of BW	1.45 ± 0.05	1.74 ± 0.06^*∗*^	1.38 ± 0.14^#^	1.19 ± 0.08^#^

Values were expressed as mean ± S.E. (*n* = 10). ^*∗*^ *P* < 0.05 versus Cont.; ^#^ *P* < 0.05 and ^##^ *P* < 0.01 versus HF. BW: body weight; Cont.: control; HF: high fructose; BC1: blackcurrant 100 mg/kg/day; BC2: blackcurrant 300 mg/kg/day.

**Table 2:** Effects of BC on CRP, T-Bil, leptin, and insulin.

	Cont.	HF	BC1	BC2
CRP (ng/mL)	195.62 ± 1.32	208.82 ± 5.6^*∗*^	194.1 ± 2.52	193.67 ± 2.39^#^

T-Bil (mg/dL)	0.6 ± 0.03	0.72 ± 0.05^*∗*^	0.63 ± 0.04	0.57 ± 0.02^#^

Leptin (pg/dL)	0.62 ± 0.03	0.45 ± 0.03^*∗*^	0.53 ± 0.01	0.83 ± 0.1^##^

Insulin (mg/dL)	0.64 ± 0.03	1.15 ± 0.19^*∗*^	0.64 ± 0.06^#^	0.62 ± 0.05^#^

Values were expressed as mean ± S.E. (*n* = 10). ^*∗*^ *P* < 0.05 versus Cont; ^#^ *P* < 0.05 and ^##^ *P* < 0.01 versus HF. CRP: C-reactive protein; T-Bil: total bilirubin; Cont.: control; HF: high fructose; BC1: blackcurrant 100 mg/kg/day; BC2: blackcurrant 300 mg/kg/day.

**Table 3:** Lipid profile in SD rats fed HF and/or BC for 8 weeks.

	Cont.	HF	BC1	BC2
T-Cho (mg/dL)	55.36 ± 1.65	73.66 ± 3.62^*∗∗*^	59.93 ± 3.07^#^	58.36 ± 3.76^#^
TG (mg/dL)	98.13 ± 13.43	229.66 ± 15.29^*∗∗*^	137.32 ± 14.42^##^	109.38 ± 13.36^##^
LDL-c (mg/dL)	31.37 ± 3.73	46.76 ± 8.85^*∗*^	33.32 ± 4.97^#^	34.35 ± 2.63^#^
HDL-c (mg/dL)	30.37 ± 4.8	27.49 ± 5.72	32.64 ± 5.2	35.1 ± 7.62

Values were expressed as mean ± S.E. (*n* = 10). ^*∗*^ *P* < 0.05 and ^*∗∗*^ *P* < 0.01 versus Cont; ^#^ *P* < 0.05 and ^##^ *P* < 0.01 versus HF. T-Cho: total cholesterol; TG: triglyceride; LDL-c: low-density lipoprotein; HDL-c: high-density lipoprotein; Cont.: control; HF: high fructose; BC1: blackcurrant 100 mg/kg/day; BC2: blackcurrant 300 mg/kg/day.
